# A curriculum for epilepsy surgery: A report from the Surgical Commission's Epilepsy Surgery Educational Task Force and the Educational Council of the ILAE

**DOI:** 10.1002/epd2.70054

**Published:** 2025-06-26

**Authors:** Christian Dorfer, Johannes M. Nico Enslin, Carrie R. Muh, Xiongfei Wang, Tatiana von Hertwig Fernandes de Olivera, Samuel Wiebe, Guy M. McKhann, Eyiyemisi Damisah, Faisal Al‐Otaibi, Gagandeep Singh, Ingmar Blümcke, Bertil Rydenhag, Rushna P. Ali, Dario J. Englot, Mario Alonso Vanegas, J. Helen Cross, Arthur Cukiert, for the Epilepsy Surgery Education Taskforce and the Educational Council of the International League Against Epilepsy

**Affiliations:** ^1^ Department of Neurosurgery Medical University of Vienna Vienna Austria; ^2^ Division of Neurosurgery, Department of Surgery University of Cape Town and Red Cross War Memorial Children's Hospital Cape Town South Africa; ^3^ Department of Neurosurgery Maria Fareri Children's Hospital, Westchester Medical Center Valhalla New York USA; ^4^ Department of Pediatrics Maria Fareri Children's Hospital, Westchester Medical Center Valhalla New York USA; ^5^ Department of Neurosurgery and Beijing Key Laboratory of Epilepsy, Epilepsy Center Sanbo Brain Hospital, Capital Medical University Beijing China; ^6^ Department of Neurosurgery Hospital Universitario Cajuru Curitiba Brazil; ^7^ Department of Clinical Neurosciences and Clinical Research Unit, Cumming School of Medicine University of Calgary Calgary Alberta Canada; ^8^ Department of Neurological Surgery Columbia University Irving Medical Center/New York Presbyterian Hospital New York New York USA; ^9^ Department of Neurosurgery, Comprehensive Epilepsy Center, School of Medicine Yale University, Yale‐New Haven Hospital New Haven Connecticut USA; ^10^ Neuroscience Center King Faisal Specialist Hospital and Research Centre Riyadh Saudi Arabia; ^11^ Department of Neurology Dayanand Medical College & Hospital Ludhiana India; ^12^ Department of Neuropathology University Hospitals Erlangen Erlangen Germany; ^13^ Department of Neurosurgery and The Sahlgrenska Academy Sahlgrenska University Hospital Gothenburg Sweden; ^14^ Department of Neurological Surgery Mayo Clinic Rochester Minnesota USA; ^15^ Department of Neurological Surgery Vanderbilt University Medical Center Nashville Tennessee USA; ^16^ Department of Biomedical Engineering Vanderbilt University Medical Center Nashville Tennessee USA; ^17^ Department of Neurosurgery National Institute of Neurology and Neurosurgery Mexico City Mexico; ^18^ UCL‐NIHR BRC Great Ormond Street Institute of Child Health London UK; ^19^ Department of Neurosurgery, Epilepsy Surgery Program Clinica Cukiert São Paulo Brazil

**Keywords:** epilepsy surgery, epilepsy surgery curriculum, epilepsy surgery education, neurosurgery curriculum

## Abstract

**Objective:**

There is a need to develop a comprehensive, formalized, and globally applicable epilepsy surgery curriculum in order to help standardize the quality of epilepsy surgery practice in the world.

**Methods:**

The Epilepsy Surgery Educational Task Force of the ILAE developed a competency‐based epilepsy surgery educational curriculum comprised of four domains: Diagnosis, Counseling, Pre‐surgical Work‐up, and Surgical Techniques. To evaluate this educational curriculum, a survey questionnaire consisting of 11 items was sent out for feedback to ILAE and IESS members. The survey questionnaire asked respondents to rate the degree of importance of each competency on a five‐point scale that ranged from “extremely important” to “not at all important” and provided space for free text comments. All competencies that were rated as “slightly important” or “somewhat important” by more than 10% of responders, as well as all free text feedback comments, were fully reviewed, and the final curriculum was adjusted accordingly.

**Results:**

One hundred and twenty‐two responses (*n* = 122) were submitted by the ILAE and IESS communities. Seventy‐one percent (71.9%) of the responses came from neurosurgeons of varied experience and training levels. Ninety‐four percent (94.4%) of responders stated that they “support a curriculum to determine the competency of neurosurgeons participating in the care of people with epilepsy in my country,” and 94.3% confirmed that they “would be interested in taking or would recommend an ILAE course based on these competencies, that would provide an ILAE certificate of completion.” Relevant changes within the curriculum were made based on feedback.

**Significance:**

The newly developed ILAE comprehensive epilepsy surgery educational curriculum has a high level of support from neurosurgeons and neurologists around the world and shows potential for broad acceptance and implementation.

**Key points**

- This is the first comprehensive epilepsy surgery curriculum established by leading neurosurgeons, neurologists and educators from around the world.
- The curriculum covers all aspects of epilepsy surgery. This is includes not only surgical techniques but aspects of clinical diagnosis, patient counseling and pre‐surgical work up.
- It reaches a high level of appreciations among neurosurgeons and neurologists.
- The curriculum represents the basis for the development of educational courses and a procedure‐ and skill based curriculum.

## 1 INTRODUCTION

The education and training of epilepsy surgeons varies significantly both nationally and globally, even in countries where access to epilepsy surgery is readily available. This variability has the potential to directly impact patient care. There is a need for an established curriculum that sets minimum standards for the education and training of epilepsy surgeons around the world.^1^

Educational and training curricula are not novel in the epilepsy field or in neurosurgery. This has been demonstrated by a recent report of the epilepsy surgery education task force of the International League Against Epilepsy (ILAE).^1^ Epilepsy surgery education and training is not following standardized protocols even in countries where access to epilepsy surgery is readily available. This lack of guidance in what a practicing epilepsy surgeon should know becomes more apparent in low‐ and middle‐income countries (LMIC), where this has greater potential to impact patient outcomes. A more standardized training framework is needed to improve patient outcomes.

A competency‐based educational curriculum for epileptologists and primary health‐care providers was recently developed by the ILAE.^2^, ^3^ Various neurosurgical subspecialties have clear curricula already established or in development. For example, the European and International Societies for Pediatric Neurosurgery (ESPN & ISPN) have successfully been training neurosurgical residents and fellows for many decades with a clear curriculum and teaching courses following a 4‐year cycle.^4^ Endovascular Neurosurgery training in Europe has recently developed a formal curriculum. Successful completion of the curriculum is mandatory before starting practice in participating centers.

Developing a core epilepsy surgery curriculum within the framework of the ILAE would help to increase the quality of epilepsy surgery; engage early‐career neurosurgeons around the world to specialize in epilepsy surgery; and may help to further demystify epilepsy surgery among physicians, patients, patient advocates, and caregivers.

## 2 METHODS

In 2021, the ILAE Surgical Commission commissioned a Task Force with the objective of addressing the need for access to and education about epilepsy surgery. The Epilepsy Surgery Education Task Force is comprised of members from the six ILAE regions, with representation across high‐income (HIC), and low‐ and middle‐income (LMIC) countries, as defined by the World Bank.^5^ All members have experience in epilepsy surgery education. Since the beginning of 2022, the Task Force met virtually to work on a core curriculum, initially focusing on a knowledge‐based curriculum, to be followed in the future by a procedure and skill‐based curriculum.

The Epilepsy Surgery Curriculum structure was framed in accordance with the Epilepsy Curriculum for epileptologists and primary health‐care providers.^2^, ^3^ Domains, competencies, and learning objectives were developed within the ILAE Educational Task Force and then approved by the ILAE Surgical Commission, followed by the ILAE Education Council. Two levels based on the criteria developed by Gaillard et al.^6^ were developed: Level 1 (basic) and Level 2 (advanced). The proposed domains and competencies were then submitted for review and commentary through an open online survey sent to both the ILAE and International Epilepsy Surgery Society (IESS) communities through open web, social media, and email (1800 persons; IESS mailing list), as recommended by the ILAE Educational Council. The survey questionnaire consisted of 11 items asking respondents to rate the degree of importance of each competency on a five‐point scale that ranged from “extremely important” to “not at all important”, supplemented by the opportunity to provide free text comments. Additionally, six questions asked for general feedback:

- Is there a competency missing that you think is relevant for the appropriate care of people with epilepsy by neurosurgeons?
- Do you have a specific epilepsy surgery education program in your country?
- Can you identify any barriers that may prevent using this educational curriculum in your country?
- Would you support this curriculum to determine the competency of neurosurgeons participating in the care of people with epilepsy in your country?
- Would you be interested in taking or would you recommend an ILAE course based on these competencies that would provide an ILAE certificate of completion?
- Would you follow this curriculum in your own practice?

The survey utilized the SurveyMonkey platform and remained open from August 3rd, 2024, to October 20th, 2024. The Task Force reviewed feedback from the survey: All components that were rated as “slightly important” or “somewhat important” by more than 10% of the responders were fully reviewed, as were all free text feedback responses. Based on the survey feedback, key changes were incorporated to create the final version of the Epilepsy Surgery Curriculum. The final curriculum was rediscussed and approved by the ILAE Educational Council and sent to the ILAE Executive Committee.

## 3 RESULTS

The final version of the Epilepsy Surgery Curriculum comprises four domains: Diagnosis, Counseling, Presurgical Work‐up, and Surgical Techniques. The Domain Diagnosis has 5 competencies with 21 learning objectives; Counseling contains 2 competencies with 7 learning objectives; Presurgical Work‐up has 7 competencies and 15 learning objectives, and Surgical Techniques has 13 competencies and 71 learning objectives (Table 1).

**TABLE 1:** Domains, competencies, and learning objectives of the ILAE and IESS epilepsy surgery curriculum.

**Domains:**
**1.0 Diagnosis**
**Competencies:**
**1.1 Demonstrate working knowledge of etiologies for focal and generalized epilepsies in children and adults**
***Learning objectives:***
1.1.1 Describe the common structural etiologies amenable to surgery (e.g., hippocampal sclerosis, tumors, malformations of cortical development, vascular lesions, traumatic brain injury, etc.) (L1)
1.1.2 Describe other common etiologies (e.g., genetic, infectious, metabolic, immune, neurodegenerative causes) (L1)
1.1.3 Demonstrate knowledge on the impact of the etiology of epilepsy on surgical prognosis (L2)
**1.2 Identify and describe seizure semiology using standardized ILAE terminology and classification systems**
***Learning objectives:***
1.2.1 Recognize the semiology of epileptic seizures and distinguish it from the semiology of non‐epileptic events (L1)
1.2.2 Extract semiological information from patient history (L1)
1.2.3 Extract semiological information from video recordings (L1)
1.2.4 Interpret semiological signs and symptoms to form hypotheses on the localization of focal seizures (L2)
1.2.5 Interpret semiological signs and symptoms that distinguish focal vs. generalized seizure onset (L2)
**1.3 Accurately diagnose and classify epilepsies and epilepsy syndromes using the most recent ILAE classifications**
***Learning objectives:***
1.3.1 Demonstrate knowledge regarding candidacy for epilepsy surgery based on epilepsy and syndrome classification (L1)
1.3.2 Correctly diagnose focal epilepsies (L1)
1.3.3 Correctly diagnose generalized epilepsies (L1)
1.3.4 Correctly classify focal epilepsies (L2)
1.3.5 Correctly classify generalized epilepsies (L2)
**1.4 Describe basic EEG patterns in children and adults**
***Learning objectives:***
1.4.1.1 Determine which patients benefit from EEG based on its diagnostic yield (L1)
1.4.1.2 Interpret EEG reports and apply them to specific clinical contexts (L1)
1.4.2 Recognize and describe the main types of EEG recordings (e.g. routine scalp, ambulatory, video‐EEG, intracranial EEG) (L1)
1.4.3 Recognize and describe the basic epileptiform and non‐epileptiform interictal abnormalities on scalp EEG (L1)
1.4.4 Recognize and describe the common ictal patterns on scalp EEG (L2)
1.4.5 Recognize and describe the common ictal and interictal patterns on intracranial EEG recordings (L2)
**1.5 Accurately interpret neuroimaging as it pertains to epilepsy**
***Learning objectives:***
1.5.1 Recognize and describe the optimum MRI sequences recommended for epilepsy (L1)
1.5.2 Define, interpret and apply the results of specialized neuroimaging accurately in the clinical context (e.g., functional, metabolic, postprocessing, etc) (L1)
**2.0 Counseling**
**Competencies:**
**2.1 Understand and address the culturally appropriate aspects and consequences of the diagnosis of epilepsy, including stigma, and its implications for pre‐surgical and surgical decision‐making**
**2.2 Provide guidance on the surgical treatment of epilepsy**
***Learning objectives:***
2.2.1 Communicate to patients and families the concept of drug‐resistant epilepsy (L1)
2.2.2 Communicate to patients and family the concept and rationale of candidacy for epilepsy surgery (L1)
2.2.3 Communicate to patients and family the rationale for different types of surgery for epilepsy (L1)
2.2.4 Communicate to patients and family the rationale, risks and benefits of intracranial EEG (L1)
2.2.5 Provide guidance to patients and family regarding risks and benefits of epilepsy surgery (L1)
2.2.6 Communicate to patients and families the benefits of timely surgical intervention (L1)
2.2.7 Identify and provide counseling on issues related to comorbidities as they may impact epilepsy surgery (L1)
**3.0 Pre‐surgical evaluation**
**Competencies:**
**3.1 Demonstrate working knowledge of the indications for pre‐surgical evaluation**
***Learning objectives:***
3.1.1. Define drug‐resistant epilepsy and how to establish drug‐resistance and drug‐responsiveness (L1)
3.1.2. Demonstrate knowledge regarding candidacy for epilepsy surgery (L1)
3.1.3. Demonstrate knowledge of semiology, interpret results, and apply them to surgical decisions (L2)
3.1.4 Demonstrate knowledge of video‐EEG, interpret results and apply them to surgical decisions (L2)
3.1.5 Demonstrate knowledge of advanced structural and functional imaging, interpret results, and apply them to surgical decisions (e.g., functional MRI, SPECT, PET) (L2)
3.1.6 Demonstrate knowledge of specialized imaging methods (L2)
3.1.7 Demonstrate knowledge of neuropsychological testing, interpret results, and apply them to surgical decisions (L2)
**3.2 Describe the importance of early surgical intervention regarding seizure control, neuro‐developmental, cognitive, behavioral, and social integration outcomes**
**3.3 Demonstrate knowledge of brain and vascular anatomy including structural and functional connectivity**
***Learning objectives:***
3.3.1 Demonstrate knowledge of cortical anatomy and white matter pathways. (L1)
3.3.2. Demonstrate knowledge of the gross anatomical substrates of seizure onset and seizure propagation (e.g., mesial temporal structures, cortical areas, white matter pathways) and surrounding structures. (L1)
**3.4 Demonstrate knowledge of neuropsychological evaluation, including language and memory lateralization**
***Learning objectives:***
3.4.1 Demonstrate knowledge about the impact of surgery on neuropsychological outcomes (L1)
3.4.2. Demonstrate knowledge of surgical approaches to address risks of neuropsychological deficits (L2)
**3.5 Demonstrate working knowledge on aspects of structural and functional imaging as they pertain to epilepsy surgery**
***Learning objectives:***
3.5.1 Recommend and interpret the main functional neuroimaging modalities as they pertain to epilepsy surgery (functional MRI, interictal and ictal SPECT, and PET) (L1)
3.5.2. Interpret the results of structural and functional imaging with regard to the surgical approach and risks of surgery (L1)
**3.6 Demonstrate working knowledge of evaluation of intracranial EEG (depth and subdural electrodes)**
***Learning objectives:***
3.6.1 Understand and describe the risks, benefits, and indications of depth and subdural electrodes (L1)
3.6.2 Demonstrate ability to integrate results of the intracranial EEG evaluation with anatomical data to advice on the surgical plan (L2)
**3.7 Demonstrate knowledge of the importance of the multidisciplinary team in epilepsy surgery and the role of individual team members**
**4.0 Surgical techniques**
**Competencies:**
**4.1 Demonstrate knowledge of surgical techniques for implantation of subdural EEG electrodes**
***Learning objectives:***
4.1.1 Demonstrate knowledge and the ability to choose different types of subdural EEG electrodes (L2)
4.1.2. Demonstrate the ability to discuss surgical details and techniques in the implantation of subdural EEG electrodes (L2)
4.1.3 Demonstrate knowledge about the risks of using subdural EEG electrodes (L2)
4.1.4 Demonstrate knowledge of the appropriate medical and surgical management of complications of subdural EEG electrodes (L2)
**4.2 Demonstrate knowledge of surgical techniques for implantation of depth electrodes (i.e., frame‐based, frameless, robot‐assisted methods)**
***Learning objectives:***
4.2.1 Demonstrate knowledge about different methods of depth EEG electrode implantation (L2)
4.2.2 Understand and describe advantages and limitations of different implantation methods for depth EEG electrodes (L2)
4.2.3. Demonstrate the ability to discuss surgical details in the implantation of depth EEG electrodes (L2)
4.2.4 Demonstrate knowledge about the risks of using depth EEG electrodes (L2)
4.2.5 Demonstrate knowledge of the appropriate medical and surgical management of complications of depth EEG electrodes (L2)
**4.3 Demonstrate knowledge of invasive mapping and monitoring of motor and language areas extraoperatively and intraoperatively**
***Learning objectives:***
4.3.1 Demonstrate knowledge about the advantages and disadvantages of extraoperative monitoring of motor and language function (L2)
4.3.2 Demonstrate knowledge about the advantages and disadvantages of intraoperative monitoring of motor and language function (L1)
4.3.3 Demonstrate knowledge about the techniques of cortical and subcortical white matter pathways mapping using monopolar or bipolar stimulation (L1)
4.3.4 Demonstrate knowledge about the application and interpretation of techniques to map the central sulcus, including the phase reversal technique (L2)
4.3.5 Demonstrate knowledge of extraoperative language mapping techniques as well as their advantages and limitations (L2)
4.3.6 Demonstrate knowledge of intraoperative language mapping techniques as well as their advantages and limitations (L1)
**4.4 Comprehend advantages and limitations of electrocorticography**
***Learning objectives:***
4.4.1 Demonstrate knowledge of the indications for electrocorticography (L1)
4.4.2 Demonstrate knowledge about the effect of general anesthetics on electrocorticography (L1)
4.4.3 Demonstrate ability to interpret electrocorticographic findings as they pertain to the surgical strategy (L1)
**4.5 Describe differences between disconnection versus resection techniques**
***Learning objectives:***
4.5.1 Describe the rationale for performing disconnection versus resection techniques (functional versus anatomical hemispherectomy, hemispherotomy, temporo‐parieto‐occipital disconnection versus resection) (L1)
4.5.2 Describe the potential limitations of disconnection techniques (L2)
**4.6 Demonstrate working knowledge of indications, technique, outcome, and complications of resective surgery for temporal lobe epilepsy (including selective and non‐selective approaches)**
***Learning objectives:***
4.6.1 Demonstrate knowledge of the differences of epilepsy surgery in the dominant versus nondominant temporal lobe (L1)
4.6.2 Describe and discuss the concept of selective and nonselective surgical approaches in temporal lobe epilepsy (L1)
4.6.3 Describe key surgical steps of the different types of selective resections in temporal lobe epilepsy (L1)
4.6.4 Describe key surgical steps in nonselective resections in temporal lobe epilepsy (L1)
4.6.5 Demonstrate knowledge of expected outcomes after surgical resections for temporal lobe epilepsy (L1)
4.6.6 Demonstrate knowledge of complications with different surgical approaches in temporal lobe epilepsy (L1)
**4.7 Demonstrate working knowledge of indications, technique, outcome and complications of resective extratemporal lobe surgery for epilepsy**
***Learning objectives:***
4.7.1 Describe and discuss the indications, risks, and likelihood of seizure freedom with extratemporal lobe resective surgery for epilepsy (L1)
4.7.2 Demonstrate knowledge of the surgical techniques, anatomical aspects, expected outcomes, and potential complications of frontal lobe epilepsy surgery (L1)
4.7.3 Demonstrate knowledge of the surgical techniques, anatomical aspects, expected outcomes, and potential complications of occipital lobe epilepsy surgery (L1)
4.7.4 Demonstrate knowledge of the surgical techniques, anatomical aspects, expected outcomes, and potential complications of parietal lobe epilepsy surgery (L1)
4.7.5 Demonstrate knowledge of the surgical techniques, anatomical aspects, expected outcomes, and potential complications of central lobe (rolandic) epilepsy surgery (L2)
4.7.6 Demonstrate knowledge of the surgical techniques, anatomical aspects, expected outcomes, and potential complications of epilepsy surgery of the insula (L2)
4.7.7 Demonstrate knowledge of the surgical techniques, anatomical aspects, expected outcomes, and potential complications of multilobar epilepsy surgery
**4.8 Demonstrate working knowledge on indications, technique, outcome, and complications of anatomical and functional hemispherectomy for epilepsy**
***Learning objectives:***
4.8.1 Demonstrate knowledge of the indications of anatomical and functional (disconnective) hemispherectomy for epilepsy (L2)
4.8.2 Describe key surgical steps and technical challenges involved in anatomical and functional hemispherectomy for epilepsy (L2)
4.8.3 Demonstrate knowledge of the expected outcomes after anatomical and functional hemispherectomy for epilepsy (L2)
4.8.4 Demonstrate knowledge of the surgical complications of anatomical and functional hemispherectomy for epilepsy and how to manage them (L2)
4.8.5 Demonstrate knowledge and provide advise on postsurgical care of hydrocephalus (L2)
**4.9 Demonstrate knowledge of indications, technique, outcome, and complications of hemispherotomy for epilepsy, including vertical and lateral techniques**
***Learning objectives:***
4.9.1 Demonstrate knowledge of the indications and limitations of hemispherotomy for epilepsy (L1)
4.9.2 Describe the techniques of lateral and vertical hemispherotomy (L1) in general
4.9.3 Demonstrate knowledge of the different lateral hemispherotomy techniques and discuss their rationale (L2)
4.9.4 Describe key surgical steps of the lateral hemispherotomy technique (L2)
4.9.5 Identify and describe the complications after lateral hemispherotomy (L1)
4.9.6 Describe key surgical steps of the vertical hemispherotomy technique (L2)
4.9.7 Identify and describe the complications after vertical hemispherotomy (L1)
4.9.8 Demonstrate ability to manage and advise on postoperative care after hemispherotomy (L1)
**4.10. Demonstrate knowledge of indications, technique, outcome, and complications of callosotomy for epilepsy, including anterior two thirds, posterior and complete disconnections**
***Learning objectives:***
4.10.1 Demonstrate knowledge on how to select among disconnective procedures for different epilepsy syndromes (including age, neurological and cognitive function) for which callosotomy is indicated (L1)
4.10.2 Understand and describe the different approaches for callosotomy including complete, incomplete, anterior, and posterior techniques (L1)
4.10.3 Describe key surgical steps in anterior 2/3 and complete callosotomy (L1)
4.10.4 Describe key surgical steps in posterior callosotomy (L1)
4.10.5 Demonstrate knowledge of complications of callosotomy and how to manage them (L1)
4.10.6 Describe the expected outcomes of callosotomy, including differences expected with various surgical techniques (L1)
**4.11 Demonstrate knowledge of indications, technique, outcome, and complications of neuromodulation techniques for epilepsy, including (vagus nerve stimulation, responsive neurostimulation, deep brain stimulation)**
***Learning objectives:***
4.11.1 Demonstrate knowledge of indications for different neuromodulation procedures for epilepsy (L1)
4.11.2 Describe key surgical steps for vagus nerve stimulation implantation (L1)
4.11.3 Describe the surgical steps for deep brain stimulation implantation (L2)
4.11.4 Describe the surgical steps for RNS implantation (L2)
4.11.5 Demonstrate knowledge of surgical complications and expected outcome of vagus nerve stimulation (L1)
4.11.6 Demonstrate knowledge of surgical complications and expected outcome of deep brain stimulation (L1)
4.11.7 Demonstrate knowledge of surgical complications and expected outcome of responsive neurostimulation (L1)
**4.12 Demonstrate knowledge of indications, technique, outcome, and complications of ablative procedures for epilepsy, including laser interstitial thermal therapy, radiofrequency thermocoagulation, radiosurgery, focused ultrasound**
***Learning objectives:***
4.12.1 Demonstrate knowledge about the advantages and limitations of ablative procedures for epilepsy (L1)
4.12.2 Demonstrate knowledge about the surgical technique of laser interstitial thermal therapy for epilepsy (L2)
4.12.3 Demonstrate knowledge about the surgical techniques of radiofrequency thermocoagulation for epilepsy (L2)
4.12.4 Demonstrate knowledge about the radiosurgery method for epilepsy (L2)
4.12.5 Demonstrate knowledge about the focused ultrasound method for epilepsy (L2)
4.12.6 Demonstrate knowledge about the risks and benefits of laser interstitial thermal therapy for epilepsy (L2)
4.12.7 Demonstrate knowledge about the risks and benefits of radiofrequency thermocoagulation for epilepsy (L2)
4.12.8 Demonstrate knowledge about the risks and benefits of radiosurgery for epilepsy (L2)
**4.13 Demonstrate knowledge of the anesthetic, surgical, and postoperative care differences between pediatric and adult epilepsy surgery**
***Learning objectives:***
4.13.1 Demonstrate knowledge of the hemodynamic challenges related to surgery in young children (L1)
4.13.2 Demonstrate knowledge of the differences in patient positioning and head fixation between children of different age groups and adults (L1)
4.13.3 Demonstrate knowledge of adequate postoperative care in children and adults (L1)
4.13.4 Demonstrate knowledge on how to manage surgical failures (L1)

One hundred and twenty‐two survey responses (*n* = 122) were gathered from the ILAE and IESS communities. While there was a broad representation of countries and specialties, 72% of the responses came from neurosurgeons with varying years of expertise and training levels. Ninety‐four percent of responders stated that they “support this curriculum to determine the competency of neurosurgeons participating in the care of people with epilepsy in my country,” and 94% confirmed that they “would be interested in taking or would recommend an ILAE course based on these competencies, that would provide an ILAE certificate of completion.” All competencies that were rated as “extremely important or very important” by >90% of the responders are summarized in Table 2.

**TABLE 2:** Summary of all competencies that were rated as “extremely or very important” by >90% of the responders.

	Number of respondents (%)
Domain diagnosis	
1.1 Demonstrate working knowledge of etiologies for focal and generalized epilepsies in children and adults	94.5
1.5 Accurately interpret neuroimaging as it pertains to epilepsy	96.33
Domain counseling	
2.1. Understand and address the culturally appropriate aspects and consequences of the diagnosis of epilepsy, including stigma, and its implications for pre‐surgical and surgical decision‐making	94.5
2.2 Provide guidance on the surgical treatment of epilepsy	98.17
Domain pre‐surgical work‐up	
3.1 Demonstrate working knowledge of the indications for pre‐surgical evaluation	99.08
3.2 Demonstrate working knowledge on the differences between lesional and non‐lesional epilepsy	99.08
3.3 Describe the importance of early surgical intervention regarding seizure control, neuro‐developmental, cognitive, behavioral, and social integration aspects	99.08
3.4 Demonstrate knowledge of brain and vascular anatomy including structural and functional connectivity	98.17
3.5 Demonstrate working knowledge on aspects of structural and functional imaging as they pertain to epilepsy surgery	97.25
3.6 Demonstrate knowledge of neuropsychological evaluation, including language and memory lateralization	92.66
3.7 Demonstrate working knowledge of evaluation of intracranial EEG (depth and subdural electrodes)	93.52
3.8 Demonstrate knowledge of the importance of the multidisciplinary team in epilepsy surgery and their individual roles	100
Domain surgical techniques	
4.2 Demonstrate knowledge of surgical techniques for implantation of depth electrodes (i.e., frame‐based, frameless, robot‐assisted methods)	94.44
4.3 Demonstrate knowledge of invasive mapping and monitoring of motor and language areas extraoperatively and intraoperatively	94.44
4.4 Comprehend advantages and limitations of electrocorticography	94.44
4.5 Describe differences between disconnection versus resection techniques	99.07
4.6 Demonstrate working knowledge of indications, technique, outcome and complications of resective surgery for temporal lobe epilepsy (including selective and nonselective approaches)	100
4.7 Demonstrate working knowledge on technique, outcome and complications of resective extratemporal lobe surgery for epilepsy	100
4.8 Demonstrate working knowledge on indications, technique, outcome, and complications of anatomical and functional hemispherectomy for epilepsy	94.45
4.9 Demonstrate knowledge of indications, technique, outcome, and complications of hemispherotomy for epilepsy, including vertical and lateral techniques	93.52
4.10 Demonstrate knowledge of indications, technique, outcome and complications of callosotomy for epilepsy, including anterior two thirds, posterior and complete disconnections	96.3
4.11 Demonstrate knowledge of indications, technique, outcome and complications of neuromodulation techniques for epilepsy, including (vagus nerve stimulation, responsive neurostimulation, deep brain stimulation)	95.37
4.12 Demonstrate knowledge of indications, technique, outcome and complications of ablative procedures for epilepsy, including laser interstitial thermal therapy, radiofrequency thermocoagulation, radiosurgery, focused ultrasound	91.67
4.13 Demonstrate knowledge of the anesthetic on surgical and postoperative care differences between pediatric and adult epilepsy surgery	92.60

Only four competencies were rated as “somewhat important” or “slightly important” by more than ten percent of the responders (Table 3).

**TABLE 3:** Summary of all competencies that were rated as “somewhat important” or “slightly important” by >10% of the responders.

	Number of respondents (%)
Domain diagnosis	
1.2 Identify and describe seizure semiology using standardized ILAE terminology and classification systems	11.28
1.3 Accurately diagnose and classify epilepsies and epilepsy syndromes using the most recent ILAE classifications	17.43
1.4 Describe common EEG patterns in children and adults	35.78
Domain surgical techniques	
1.1 Demonstrate knowledge of surgical techniques for implantation of subdural EEG electrodes	13.90

The low importance feedback for Domain Diagnosis 1.2 and 1.3 regarding identification and description of seizure semiology as well as diagnosing and classification of epilepsies using the most recent ILAE classifications was discussed and re‐evaluated by the Task Force, but felt to be important to stimulate interdisciplinary appreciation and therefore remains. To address feedback for Domain Diagnosis 1.4 stating that “Describe common EEG patterns in children and adults” was of low importance, we modified the curriculum to “Describe basic EEG patterns…”. In parallel, we adjusted the associated learning objectives to the ability to recognize basic (instead of common) epileptiform and non‐epileptiform interictal abnormalities (1.4.3), as well as to recognize main ictal patterns on scalp and intracranial recordings, instead of common patterns (1.4.4 and 1.4.5). Regarding the low importance rate for knowledge of surgical techniques for implantation of subdural EEG electrodes, the Task Force felt that even though depth electrode placement has become the method of choice for invasive EEG in most centers, subdural electrode evaluations still have its role in selected cases and are also being used by centers where depth electrode placement is not possible for given reasons. Therefore, this competency remained included in the final curriculum.

Based on the free text feedback, we added one additional learning objective for the competency 4.13—demonstrate knowledge of the anesthetic, surgical and postoperative care differences between pediatric and adult epilepsy surgery, so that it now includes the learning objective “Demonstrate knowledge on how to handle surgical failures” (4.13.4).

## 4 DISCUSSION

The newly developed Epilepsy Surgery Curriculum represents a comprehensive description of the educational and training milestones that trainees should target and practicing epilepsy surgeons should have or work to acquire. In contrast to previous, more limited curricula, it covers all important aspects of providing comprehensive care for surgical epilepsy patients.^7^, ^8^, ^9^ This goes beyond knowledge of surgical techniques and includes aspects of clinical diagnosis, patient counseling, as well as an in depth understanding of the methods and interpretation of the results obtained in the pre‐surgical work‐up. This should enable epilepsy surgeons to actively take part in the multidisciplinary epilepsy team discussions rather than having a more passive presence. Adequately incorporating neurosurgical perspectives into the multidisciplinary discussions would likely increase the quality of decision‐making and care for epilepsy surgery patients and caregivers.^10^ Moreover, while many of the different surgical techniques might not be available in individual centers, knowledge about the methods, advantages and disadvantages, risks, and benefits as well as potential impact on outcome is crucial.

It is important for epilepsy surgeons to understand the language and methods utilized by our epileptology colleagues, just as it is important for neurologists to have a fundamental understanding of epilepsy surgical anatomy and procedures. For this reason, the competencies 1.1—identify and describe seizure semiology using standardized ILAE terminology and classification systems and 1.2—accurately diagnose and classify epilepsies and epilepsy syndromes using the most recent ILAE classifications—were kept unchanged after thorough discussion, even though their rates did not reach a high importance level on the questionnaire feedback.

In many countries and institutions, this advanced comprehension of epilepsy surgery is already being practiced, but it is lacking in many others. Systematic epilepsy surgery education through a standardized curriculum as presented may help to advance the field and ensure that the best possible quality of care is delivered to patients. It also may facilitate a better understanding of the neurosurgeon's role within the multidisciplinary epilepsy team. Epilepsy surgery planning and execution is fundamentally built on teamwork and flourishes when there is understanding and appreciation of each member's specific contributions.

Nevertheless, there may be barriers to implementing such a curriculum that need to be identified, discussed, and elaborated upon. One of these barriers is cost. Neurosurgeons from low‐income countries may find it difficult to attend courses or get educational material for which financial support would be needed. Others might need to travel to find or identify mentors. Some equipment or imaging technologies that are readily available in high‐income countries may be more difficult to obtain in LMICs, and some hospital systems may not have full‐time multidisciplinary teams including neuroradiologists or neuropsychologists. Likewise, in countries with centralized care, access to these may be much easier. Implementation or adjustment of a formalized curriculum may also be complicated by costs derived from paperwork, management, and monitoring processes. As the curriculum presented here is knowledge‐based, many of these obstacles may be addressed through online educational material and courses. Continuous financial support, however, would be mandatory as these efforts need technical support and educators with the willingness and ability to dedicate time to educational programs. This has succeeded for many educational activities among other neurosurgical subspecialties, where specific curricula, similar to the one described here, were applied.^4^ The presented curriculum thus might represent a valuable instrument to set common standards in surgery for epilepsy once it is widely disseminated and accepted among neurosurgeons and neurologists involved in the treatment of epilepsy.

The next steps in moving this curriculum project forward will be the development of a procedure‐ and skill‐based curriculum. Such a curriculum may help to further define different levels of epilepsy surgery centers for education and practice. The major challenge here lies in defining the different levels of experience and acumen within the various types of epilepsy surgery procedures, which are continuously evolving. One framework could encompass a two or three‐level curriculum (entry, proficiency, advanced). The competencies, knowledge, and skillsets attributed to these levels may not be easily defined; however, there are major practice discrepancies around the world, and team development and care pathways should be further discussed as a next step within a multidisciplinary taskforce.

## 5 CONCLUSION

This is the first comprehensive epilepsy surgery curriculum established by leading neurosurgeons from around the globe and supported by leading neurologists and educators. It reached a very high rate of appreciation among neurosurgeons and neurologists in our questionnaire feedback and holds promise to be broadly accepted and implemented worldwide.

## FUNDING INFORMATION

No funding was received for this work.

## CONFLICT OF INTEREST STATEMENT

The authors declare no conflict of interest.

6

**Test yourself**

1. Which competencies were rated as not so important for epilepsy surgeons?
  - Demonstrate knowledge of etiologies for focal and generalized epilepsies
  - Accurately interpret neuroimaging as it pertains to epilepsy
  - The ability to describe common EEG patterns
  - Demonstrate working knowledge of the indications for presurgical evaluation
2. Which competency in the Domain Surgical Techniques was rated as not important but remained unchanged after re‐discussion within the Educational Task Force and the Educational Council
  - Demonstrate knowledge of the surgical techniques for implantation of subdural EEG electrodes
  - Comprehend advantages and limitations of electrocorticography
  - Demonstrate knowledge of the anesthetic, surgical, and postoperative care differences between pediatric and adult epilepsy
  - Describe differences between disconnection versus resection techniques
3. What was the percentage of responders rating the competency to describe common EEG patterns as only somewhat important?
  - 10.5%
  - 25.7%
  - 35.8%
  - 44.5%
4. How many responders rated that working knowledge of the indications for pre‐surgical evaluation is very important?
  - 89.8%
  - 100%
  - 99.0%
  - 94.6%

*Answers may be found in the* *supporting information.*

## Supporting information


Data S1

## Data Availability

The data will be provided by the corresponding author upon reasonable request.

## References

[1] Nico Enslin JM , Muh CR , Wang X , von Hertwig Fernandes de Olivera T , GM MK , Damisah E , et al. Epilepsy surgery education and practice around the globe: an ILAE taskforce report. Epilepsia. 2024;66:319–327. 10.1111/epi.18199 39636690 PMC11827745

[2] Blümcke I , Arzimanoglou A , Beniczky S , Wiebe S . Roadmap for a competency‐based educational curriculum in epileptology: report of the Epilepsy Education Task Force of the International League Against Epilepsy. Epileptic Disord. 2019;21(2):129–140. 10.1684/epd.2019.1039 30892268

[3] Singh G , Braga P , Carrizosa J , Prevos‐Morgant M , Mehndiratta MM , Shisler P , et al. An epilepsy curriculum for primary health care providers: a report from the Education Council of the International League Against Epilepsy. Epileptic Disord. 2022;24(6):983–993. 10.1684/epd.2022.1479 35993831

[4] The postgraduate course of the European Society for Pediatric Neurosurgery (ESPN). Available from: https://www.espneurosurgery.org/espn‐postgraduate‐course

[5] Hamadeh N , Van Romapaey C , Metreau E . World Bank country classification by income status 2020–2021. World Bank Blogs; 2021. Available from: https://blogs.worldbank.org/opendata/new‐world‐bank‐country‐classifications‐income‐level‐2021‐2022

[6] Gaillard WD , Jette N , Arnold ST , Arzimanoglou A , Braun KPJ , Cukiert A , et al. Establishing criteria for pediatric epilepsy surgery center levels of care: report from the ILAE Pediatric Epilepsy Surgery Task Force. Epilepsia. 2020;61(12):2629–2642. 10.1111/epi.16698 33190227

[7] The American Epilepsy Society (AES) core curriculum‐surgical. Available from: https://www.pathlms.com/aes/courses/7296

[8] The epilepsy fellowship curriculum of the Wake Forrest University. Available from: https://school.wakehealth.edu/education‐and‐training/residencies‐and‐fellowships/epilepsy‐fellowship/curriculu

[9] The epilepsy surgery fellowship from the Nationwide Children's Hospital. Available from: https://www.nationwidechildrens.org/for‐medical‐professionals/education‐and‐training/fellowship‐programs/epilepsy‐surgery‐fellowship

[10] Engel J Jr . Surgical treatment of epilepsy: the role of multidisciplinary care. Epilepsia. 2013;54(7):1181–1187.

