# Supplementary material for: A curriculum for epilepsy surgery: A report from the Surgical Commission's Epilepsy Surgery Educational Task Force and the Educational Council of the ILAE
Source: Epileptic Disord. 2025 Jun 26;27(5):718–28. doi: 10.1002/epd2.70054 (PMC12574484; doi:10.1002/epd2.70054)
Supplement: Supplementary file 1 — Data S1 [file EPD2-27-718-s001.docx]

**TEST YOURSELF**

**Answers:**

1. (C)
2. (A)
3. (C)
4. (C)
